# Supplementary material for: Disulfiram (Tetraethylthiuram Disulfide) in the Treatment of Lyme Disease and Babesiosis: Report of Experience in Three Cases
Source: Antibiotics (Basel). 2019 May 30;8(2):72. doi: 10.3390/antibiotics8020072 (PMC6627205; doi:10.3390/antibiotics8020072)
Supplement: Supplementary file 1 [file antibiotics-08-00072-s001.zip › Supplementary 2b Republication Auth.pdf]

To Whom it May Concern:

I, Karen Vanderhoof- Forschner, J.D., authorize the re-publication in print and/or electronic media of the following article, authored by Kenneth B. Liegner et. al.:

Lyme disease and the Clinical Spectrum of Antibiotic Responsive Chronic Meningoencephalomyelitides.

The Journal of Spirochetal and Tick-borne Diseases retains copyright.

Karen Vanderhoof-Forschner

Karen Vanderhoof Forschner — Date

Nov 2, 2018
