# Supplementary material for: Disulfiram (Tetraethylthiuram Disulfide) in the Treatment of Lyme Disease and Babesiosis: Report of Experience in Three Cases
Source: Antibiotics (Basel). 2019 May 30;8(2):72. doi: 10.3390/antibiotics8020072 (PMC6627205; doi:10.3390/antibiotics8020072)
Supplement: Supplementary file 1 [file antibiotics-08-00072-s001.zip › Supplementary 1 Abstracts for Remission mss.pdf]

V International Conference
on Lyme Borreliosis

PROGRAM AND ABSTRACTS

Arlington, Virginia, U.S.A.
May 30-June 2, 1992
Hyatt Regency Crystal City

**63**

## CULTURE-CONFIRMED TREATMENT FAILURE OF CEFOTAXIME AND MINOCYCLINE IN A CASE OF LYME MENINGOENCEPHALOMYELITIS IN THE UNITED STATES.

Kenneth B. Liegner, Carl E. Rosenkilde, Grant L. Campbell*, Thomas J. Quan, and David T. Dennis, Armonk, NY, USA, Mount Kisco, NY, USA, and Centers for Disease Control, Fort Collins, CO, USA.

In 1987, a 37-year-old woman living in Westchester County, NY, developed spastic paraparesis, bilateral Babinski reflexes, and cranial nerve and bulbar dysfunction characterized by dysphagia, dysphonia, diplopia, absent gag reflex, and dysfunction of bowel and bladder control. CSF contained 19 WBC/mm$^3$ (86% lymphs). A test for antibodies to *Borrelia burgdorferi* (*Bb*) in serum was negative. No etiology was established despite an extensive workup. Symptoms and signs reportedly worsened gradually from 1988 to present. There was a past history of splenectomy for idiopathic thrombocytopenic purpura diagnosed in 1975. In 1989, the right frontal region and right basal ganglia were abnormal on brain MRI. In January 1990, CSF contained 6 WBC/mm$^3$ (93% lymphs), but no oligoclonal bands or myelin basic protein. Paired CSF and serum tests for antibodies to *Bb*, and PCR for *Bb*-specific oligonucleotides in CSF, were negative. An empiric 21-day course of cefotaxime (3 g/12 hr i.v.) was given in January, 1990 with no clear clinical benefit. Following treatment, CSF contained 9 WBC/mm$^3$ (93% lymphs). Four months of minocycline (200 mg/day p.o.) begun in November, 1990 also yielded no clear clinical benefit. In December, 1990 a T-cell stimulation test with *Bb* antigens was strongly positive. In December, 1991 CSF contained 6 WBC/mm$^3$ (89% lymphs) and elevated IgG. Paired serum and CSF samples were strongly positive for antibodies to *Bb*, with a CSF-to-serum index of 1.04. Culture of this CSF specimen in BSK-II yielded a strain of *Bb*. Culture-confirmed treatment failures have been previously reported for three Lyme neuroborreliosis cases in Europe. The present case apparently is the first of this type to be reported from the United States.

VI INTERNATIONAL CONFERENCE ON LYME BORRELIOSIS

Bologna, Italy, June 19-22, 1994

PROGRAM AND ABSTRACTS

# P012M

## CHRONIC LYME DISEASE (CLD): A COSTLY DILEMMA.

Liegner KB(1), Agricola MD(1), Bayer ME(2), Duray PH(3).
Private practice, Armonk, N.Y.(1), Fox Chase Cancer Center, Philadelphia, PA(2), Brigham & Women's Hospital, Boston, MA(3).

B. burgdorferi (Bb) was isolated from the CSF of a 41 year-old woman with previously seronegative chronic meningoencephalomyelitis (CMEM), spastic tetraparesis and cranial nerve pareses of at least 3 years duration on 12/91, 6 months following empiric treatment with 21 days of I.V. cefotaxime (CFOTX) and 4 months of minocycline. After the diagnosis was proven she was treated with "pulse" CFOTX from January 1992 until December 1992. Pleocytosis, chronic fatigue, and hypersomnolence resolved but neurologic status continued to deteriorate very gradually and inappetance developed. Re-emergence of minimal pleocytosis was noted. Because of increasing spasticity unresponsive to maximal doses of dantrolene and oral baclofen, she was transferred to a tertiary care facility for rehabilitation and consideration of implantation of a programmable pump for continuous intra-thecal infusion of baclofen. Two weeks of daily ceftriaxone were given to "assure" complete eradication of any residual infection, and then treatment with "pulse" methylprednisolone for 5 days was given followed by 60 mg/day prednisone for some six weeks on the premise that her neurologic illness was due to a primary autoimmune diathesis. This resulted in no clinical benefit and refractory bilateral pleural and pericardial effusions developed which were attributed to a "lupus-like" illness, as complement was reported depressed. 50 micrograms of baclofen instilled intrathecally resulted in near total limb paralysis for 24 hours; further attempts to titrate baclofen dose to effect, were abandoned. The patient was transferred back to the referring physician. Antibiotics were withheld for a total of some 5 months. Severe encephalopathy and slurring of speech developed. She was unable to hold a cup, roll over in bed, or transfer to a wheel chair. Sedimentation rate was greater than 100 mm/hour. Aspiration pneumonia developed. Routine and TBC smears and cultures of pleural fluid were negative. Pleural fluid complement was normal. CSF showed 5 mono-nuclear cells/mm3. An experimental PCR assay for the plasmids encoding for OspA was positive on pleural and cerebrospinal fluids. Pericardial window was performed for diagnostic and therapeutic purposes. Fibrinous pericarditis was found. Two separate spirochete-compatible forms were found on silver stain histopathology, with an accompanying infiltrate of mononuclear and plasma cells. Intravenous CFOTX and clindamycin were begun, the latter for treatment of the aspiration pneumonitis. CFOTX was given for 109 continuous days. During this time encephalopathy gradually cleared with remarkable improvement in mental status and motor strength, the patient able to walk 500 feet with a rolling walker. Pericardial and pleural effusions resolved almost completely as assessed by serial CT scans of the chest. ESR decreased to the range of 40-60 mm/hr, and normal appetite returned. A 15 microgram bolus of intrathecal baclofen resulted in significant reduction in spasticity enabling the patient to widely separate knees that had been in spastic apposition, with transiently improved limb mobility and gait. Attempted reduction in the intensity of antibiotic therapy by using "pulse" CFOTX with daily azithromycin resulted in gait deterioration, and noticeable worsening of memory and speech slurring within two weeks. The patient was discharged 10/93 on daily CFOTX by programmable pump with a fully implanted vascular access device. At the present time (1/94) there are no plans to discontinue daily intravenous antibiotic treatment. Mental status continues to improve. Recent CXR was clear. Elective implantation of a programmable pump for continuous intra-thecal baclofen infusion is planned for the near future. We have been unable to design a regimen of oral antibiotics which can keep the patient compensated. CONCLUSION: Bb can produce chronic CNS and deep-seated systemic tissue infection which can be controlled but not eradicated with intensive long-term antibiotic treatment. This is a costly dilemma in terms of expenditure of health care resources and damaged human lives. Better means of diagnosis, prevention, and treatment of CLD are urgently needed.

# 13TH INTERNATIONAL SCIENTIFIC CONFERENCE ON LYME DISEASE & OTHER TICK-BORNE DISORDERS

MARCH 25 & 26, 2000 Farmington, CT, USA

**Poster Presenter Registration:** $160. Includes presentations, poster displays, 2 lunches, 4 breaks, Conference Compendium, 2 receptions (3/24 & 3/25).

**Room** $79 single/double **Receptions** 3/ 24 & 25 **Presentations** 3/25 & 26

## Abstract Form

**Deadline: Speaker Abstracts due 2/25/00 Poster Submissions 2/25/00**

Abstracts must be in English and you should use a separate form for each submission.

Type your abstract within the area shown. Type the title in capital letters, list authors, affiliation and location where research was done, and use an asterisk to indicate the poster presenter. Additional pages are allowed, but remember to use a left hand 1" border.

Each abstract should contain: objectives of the research, methodology, result, and conclusion. A conference committee member will contact you for more information, if needed.. Abstracts are published in the Conference Program Book, which is distributed to all conference registrants. *Abstracts are printed as submitted by the authors.* Speakers can email their abstract to us.

DISEASE LYME BORRELIOSIS

PREVENTION & BASIC SCIENCE
- __ Ecology, Entomology
- __ Vaccine
- __ Animal models
- __ Microbiology
- __ Pathogenesis
- __ Pathology
- __ Other:

PATIENT MANAGEMENT
- __ Clinical manifestations
- __ Laboratory diagnosis
- __ Early disease management
- __ Late disease management
- √ Chronic disease management
- __ Other:

**OPEN-ENDED HIGH DOSE LONG-ACTING AMOXICILLIN: AN ALTERNATIVE IN THE TREATMENT OF CHRONIC AND NEUROLOGIC LYME DISEASE.**

**Liegner KB. Private Practice. Armonk, NY. Neurologic sequelae are increasingly recognized as the major morbidity of Lyme disease. Many lines of evidence indicate that Lyme borreliosis can be a chronic infection which is suppressed but not cured with application of antibiotic therapy. Faber and Morrison each described successful treatment of neurosyphilis using high dose oral amoxicillin combined with probenicid (2 grams amoxicillin + 0.5 gram probenicid p.o. Q 8 hr) and demonstrated treponemicidal levels of amoxicillin in cerebrospinal fluid. In patients of average weight, such a regimen produces peak antibiotic blood levels measured 90 minutes after a dose of 15-25 micrograms/ml. Recently, SmithKline Beecham has introduced a long-acting preparation of amoxicillin (Amoxil 875 mg.) designed for Q 12 hour administration. This formulation can be used without resort to probenicid to achieve comparable blood levels 2 ½ hours after a dose. Dosages necessary to achieve such blood levels vary from 3 X 875 mg. up to 10 X 875 mg. p.o. Q 12 hours in persons weighing from 120 lb. up to 200 lb., respectively. Clinical response with this relatively inexpensive regimen has been excellent with good tolerability and an excellent safety profile. Many patients, including many with serious chronic neurologic involvement as determined by findings on one or more of clinical evaluation, brain SPECT, brain MRI, detailed neuropsychological testing, and cerebrospinal fluid examination have done extremely well with this regimen allowing some to avoid resort to intravenous antibiotic therapy and others to successfully seque off of intravenous antibiotics.**

**Refs:Faber WR et.al. Treponemicidal Levels of Amoxicillin in Cerebrospinal Fluid After Oral Administration. Sexually Transmitted Diseases.198;10:148-150 & Morrison RE et.al. Oral Amoxicillin, an alternative for neurosyphilis. Genitourin Med 1985;61:359-62.**

Name KENNETH B. LIEGNER MD
phone 914 273 2121
Title PHYSICIAN
fax 914 2734801
Affiliation PRIVATE PRACTICE
e-mail 0
Street 8 BARNARD RD
Signature Kenneth B Liegner MD
City ARMONK State NY Zip 10504
Country USA

Return Original by mail to:
Lyme Disease Foundation, 1 Financial Plaza, Hartford, CT 06103 860-525-2000

VI INTERNATIONAL CONFERENCE ON LYME BORRELIOSIS

Bologna, Italy, June 19-22, 1994

# P041T

FATAL CHRONIC MENINGOENCEPHALOMYELITIS (CMEM) WITH MASSIVE HYDROCEPHALUS, IN A NEW YORK STATE PATIENT WITH EVIDENCE OF BORRELIA BURGDORFERI (Bb) EXPOSURE.

Liegner KB(1), Ziska M(2), Agricola MD(1), Hubbard JD(3), Klempner MS (4), Coyle PK (5), Bayer ME (6), Duray PH(7).
Private practice, Armonk, NY (1), Lyme Disease Foundation, Hartford, CT (2), Bender Laboratory, Albany, NY (3), Tufts University School of Medicine, Boston, MA (4), SUNY School of Medicine, Stony Brook, NY (5), Fox Chase Cancer Center, Philadelphia, PA (6), Brigham & Women's Hospital, Boston, MA (7).

In the Fall of 1985 a 61 y.o. outdoorsman, a resident of Greene County in the Catskills, New York, developed a large left groin rash compatible with erythema migrans (EM). The significance of the eruption was not recognized and no treatment was given. Fatigue and headache developed and were unrelenting by 3/86. Severe myalgias, painful paresthesias, photophobia, and low grade pyrexia developed. Several CSF examinations showed lymphocytic pleocytosis with elevated protein. A progressive stroke syndrome evolved beginning 5/86. Lyme disease (LD) was discounted because serologic testing was negative. Despite prednisone and cytoxan for presumptive vasculitis, the patient deteriorated. 1988 Lyme ELISA was reactive (Stony Brook). Ceftriaxone (CFTRX) was given for 14 d I.M. with some improvement, however the patient remained bed-bound in a primitive near-vegetative neurologic state with grunting emotive vocalizations and intermittent response to simple commands for the next 4 years. When re-evaluated in 5/92, CT scan of the brain showed massive hydrocephalus. EEG showed status epilepticus, and phenobarbital was given. CSF examination at this time showed normal opening pressure, glucose and protein. Paired CSF & serum Lyme ELISAs were negative (Stony Brook) although serum ELISA was positive from CDC, Fort Collins. OCBs were present in CSF and absent in serum. MBP was absent. CSF IgG and IgG synthesis rate were markedly elevated,[(17.2 mg%;ULN 3.5) & (43.4 mg/24 hours;ULN 10.0)]. Borrelial, viral and TBC CSF cultures as well as culture and darkfield exam of conjunctival biopsy were negative. Blood abnormalities included elevated IgG ACLA, marked polyclonal IgG increase, elevated C1Q immune complexes, elevated ESR and C-reactive protein (CRP), hepatic transaminitis and anemia. Experimental PCR assay for the plasmid encoding for OspA was positive in serum and CSF. Experimental OspA antigen-capture ELISA was positive in CSF. The patient was treated with 28 d CFTRX followed by weekly "pulse" cefotaxime (CFOTX) for 11 months until 6/93. With treatment anemia and transaminitis improved, ESR and C-RP normalized, and mental and behavioral status, although severely abnormal, showed modest improvement. Serologic testing 2/93 showed reactive Lyme ELISA (Stony Brook). CFOTX was discontinued and minocycline (MNCN) given per G-tube. His status gradually deteriorated and death occurred 7/93. An autopsy showed intense lymphocytic meningoencephalomyelitis and ependymitis, with severe necrosis of neurons and astrocytes. TBC smears & cultures were negative. Spirochetes were not visualized on silver stain histopathology nor grown in culture of CNS tissues and epimyocardium. Virions were not seen on electron microscopy. Vasculitis per se was absent. OspA antigen was detected in terminal CSF.
CONCLUSION: This patient developed a severe unrelenting CMEM which ended in his death. His neurologic deterioration began following a rash consistent with EM. If this patient had active nervous system infection by Bb, corticosteroid and cytotoxic therapy for presumptive vasculitis may have contributed to the progression of his disease. LD was discounted because of initially negative serology. However, his epidemiologic exposure risk, history of rash consistent with EM, subsequent positive serologies, PCR, and antigen research assays and clinical response to antibiotics all support that this could be an unusual case of nervous system Bb infection. More aggressive early evaluation including biopsy and the application of experimental tests for LD to his CSF analysis, might have led to antibiotic treatment earlier in his course before devastating pathologic damage had occurred.

# VIII International Conference on Lyme Borreliosis and other Emerging Tick-Borne Diseases

## Munich, Germany
## June 20–24, 1999

P 380

**Fatal progressive encephalitis following an untreated deer tick attachment in a 7 year-old Fairfield County, Connecticut child.** <u>Liegner KB</u> **& Jones CR. Private Practices, Armonk, NY & Hamden, CT, USA.** An engorged deer tick was removed from the right aspect of the neck of a 6 year old Fairfield County, Connecticut girl March 1995. Parental request for prophylactic antibiotic treatment was refused by the child's physician. No eruption occurred at the tick bite site. Summer 1995 flu-like symptoms and conjunctivitis developed and October 1995, headache, stiff neck, and sleep disturbance. November 1995 right supraclavicular lymphadenitis, fever, lethargy and hypersomnolence developed. Admitted to a local hospital, focal seizures ensued. Phenytoin was administered. Lumbar puncture showed 3 white blood cells and normal glucose and protein. Phenytoin, ceftriaxone, ampicillin, and acyclovir were administered. Tests for rabies and Lyme disease were negative. MRI of brain was normal. Transfer was made to a tertiary care facility where high dose pentobarbital coma was required to control status epilepticus. Feeding gastrostomy and Broviac catheter were required for nutrition and medications. Adenovirus serology, arbovirus serology and CSF serology and culture and CSF serology, culture, and PCR for HSV-1, HSV-2, ANCA, ANA, ASO, bartonella, cold agglutinins, febrile agglutinins, influenza, parainfluenza, CSF india ink prep, malaria screen, measles, mycoplasma, Q fever, rabies, RMSF, RSV, rotavirus, rubella, toxoplasmosis, typhus, varicella, Lyme disease serologies and VDRL were negative. EBV antibodies were present. HSV IFA was positive and rose following administration of IVIG. IgG for ehrlichia was positive at 1:256 by the Centers for Disease Control. Intravenous immunoglobulins were given for putative Rasmussen's syndrome, steroids for "vasculitis", and intravenous acyclovir for the possibility of herpes encephalitis. Intravenous nafcillin was given for coagulase negative staphylococcal bacteremia. CT scans and MRIs of the brain, initially normal, demonstrated evolution of cerebral atrophy and periventricular white matter disease. June 1996 the patient demonstrated arthritis involving hands, wrists, ankles, knees, and hips, was experiencing frequent seizures and was unable to walk, speak, respond to verbal commands, or feed herself. Paired Lyme ELISAs in CSF and serum 7/96 were negative, but Lyme IgG immunoblot in serum disclosed the presence of 30, 41, 66, & 93 kiloDalton bands as well as 60 kDa band. CSF cell count, glucose, protein, and IgG were normal. CSF, blood, and urine Lyme PCRs were negative as was culture for borrelia in BSK-H. Myelin basic protein and oligoclonal bands were absent. Osp A antigen capture assay in CSF and Lyme-specific immune complexes in CSF and serum were negative. Treatment with intravenous ceftriaxone initially resulted in worsened seizure activity and treatment was changed to cefotaxime. Arthritis resolved within one month of starting intravenous antibiotics. A short course of doxycycline was given to cover the possibility of co-infection with ehrlichia. During six months of treatment with intravenous cephalosporins seizures, which had remained poorly tractable despite intensive oral anticonvulsant therapy, diminished and became readily controllable with lower dosage of anticonvulsants. The patient became able to walk, vocalize in simple sentences, feed herself, and use a swing set but remained severely neurologically impaired with significant irreversible brain injury evident on brain MRI and CT scan. Antibiotic therapy was stopped 12/3/96. Seizures reoccurred within one week of cessation of antibiotics and became increasingly difficult to manage despite continuation of anticonvulsant therapy. While in a tertiary care hospital her condition deteriorated and she died 1/30/97. An autopsy was performed.
