# Supplementary material for: Disulfiram (Tetraethylthiuram Disulfide) in the Treatment of Lyme Disease and Babesiosis: Report of Experience in Three Cases
Source: Antibiotics (Basel). 2019 May 30;8(2):72. doi: 10.3390/antibiotics8020072 (PMC6627205; doi:10.3390/antibiotics8020072)
Supplement: Supplementary file 1 [file antibiotics-08-00072-s001.zip › Supplementary 2a Remission mss - LIEGNER CLIN SPECTR CMEM JSTBD.pdf]

Lyme Disease and the Clinical Spectrum of Antibiotic Responsive Chronic Meningoencephalomyelitides

Kenneth B. Liegner, MD; Paul Duray, MD; Martha Agricola, FNP; Carl Rosenkilde, MD, PhD; Lawrence A. Yannuzzi, MD; Martina Ziska, MD; Richard C. Tilton, PhD; Dagmar Hulinska, PhD; Jeff Hubbard, MD; and Brian A. Fallon, MD

ABSTRACT

Intensive study of four patients with chronic meningoencephalomyelitis believed due to Lyme disease revealed seronegativity and/or variable seroreactivity and chronic persistent infection as common threads. Evaluation of these complex cases required determined study over time using all known methods (i.e., culture isolation, histologic, immunohistochemical, electron micrographic, direct antigen detection as well as standard serologic methods) on tissues as well as serial study of blood, cerebrospinal fluid (CSF) and urine. Prolonged intravenous antibiotic therapy conferred clinical benefit in each case and withholding of treatment resulted in clinical deterioration.

Key words: Lyme disease, meningoencephalomyelitis, persisting infection, seronegativity, *B. burgdorferi,* syphilis, multiple sclerosis, systemic lupus erythematosus

INTRODUCTION

It is commonly held that patients with late Lyme disease are almost invariably seropositive[1] and antibiotic treatment of limited duration is generally curative.[2-5] However, the phenomenology of chronic neuroborreliosis has not been fully elucidated.[6] We have encountered a significant number of patients who have been seronegative for months to years despite serious neurologic illness of long standing thought due to Lyme disease. Antibiotic treatment in these cases, while conferring benefit, has seemed unable to eradicate the infection regardless of route of administration or duration of therapy. Four such cases of chronic meningoencephalomyelitis have been extensively studied and their response to treatment carefully documented. Detailed presentation of these cases may serve to illumine this cryptic disorder, which can be as difficult to treat as it is to diagnose.

From private practices (Dr. Liegner, Nurse Agricola, Dr. Rosenkilde), Armonk and Mount Kisco, NY; National Cancer Institute (Dr. Duray), Bethesda, MD; Manhattan Eye, Ear, & Throat Hospital (Dr. Yannuzzi), New York; Lyme Disease Foundation (Dr, Ziska), Hartford, CT; BBI-North American Clinical Laboratories (Dr. Tilton), New Britain, CT; National Institute of Public Health (Dr. Hulinska), Prague, Czech Republic; Bender Laboratory (Dr. Hubbard), Albany, NY; Lyme Disease Research Program NYS Psychiatric Institute (Dr. Fallon), New York, NY.

Address correspondence to Kenneth B. Liegner, MD, 8 Barnard Road, Armonk, NY 10504.

CASE REPORTS

Case #1

A 39-year-old woman with a two-year history of progressing spastic quadraparesis, cranial nerve palsies, and persistent unexplained CSF pleocytosis was evaluated beginning in 1989. She had been diagnosed with idiopathic thrombocytopenic purpura (ITP) in 1975 and underwent splenectomy in 1976. She had lived in northern Westchester county, New York and northern California but gave no history of tick attachments or of erythema migrans.

No diagnosis was established after a year of observation and testing, and serologic studies for Lyme disease in serum and CSF were repeatedly negative. CSF examination in 1990 showed lymphocytic pleocytosis, elevated IgG, and absence of oligoclonal bands or myelin basic protein. Anticardiolipin and antinuclear antibodies were present and Raji cell assay and C1Q immune complexes were elevated. HIV and HTLV-1 antibodies were negative.

An empiric trial of intravenous antibiotic treatment with cefotaxime (CFOTX) for 21 days in April 1990 resulted in no clinical improvement and no change in CSF pleocytosis. Thereafter she was treated with 4 months of minocycline with no clinical benefit. The patient remained wheelchair-bound.

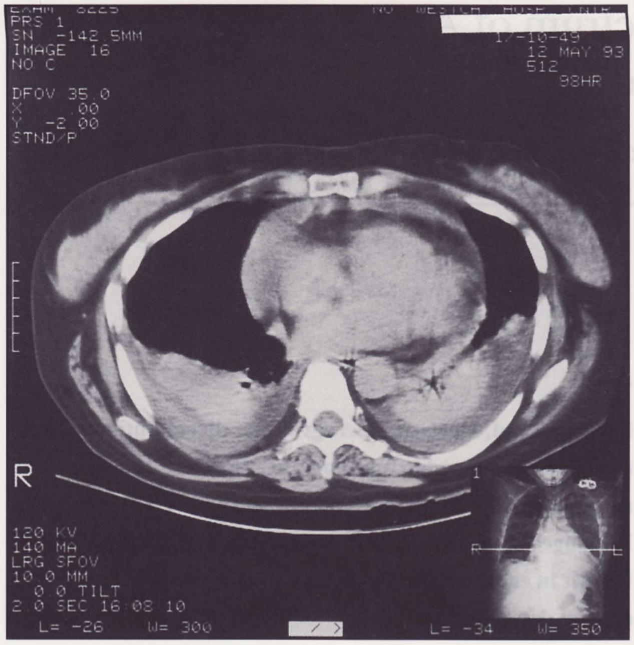

Fig 1, Case 1: Computed axial tomography of the chest showing sizable pleuropericardial effusions that developed after institution of high dose corticosteroids for the patient''s "lupus-like" illness.

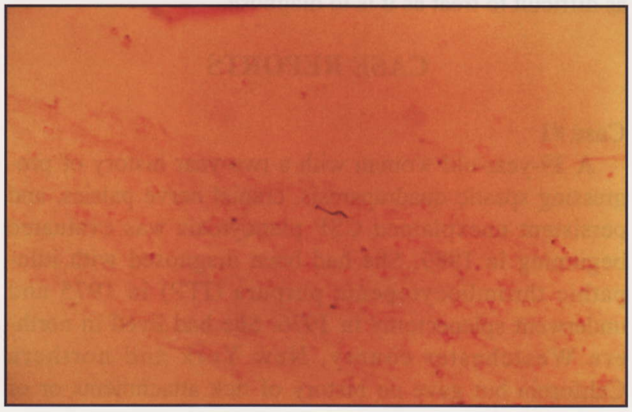

Fig 3, Case 1: Modified Steiner silver stain showing spirochete-compatible form within pericardial tissue [original magnification 1000x].

B. burgdorferi grew from CSF in December 1991 at which time the patient first became seropositive despite at least 4 years of clinical illness. She was treated with CFOTX (4 g IV Q 8 hrs once weekly) with complete resolution of pleocytosis after 13 weeks and constitutional symptoms improved. Despite continuation of once weekly IV therapy for 10 months, there was gradual neurologic deterioration. Intravenous antibiotics were discontinued December 1992.

Methylprednisolone sodium succinate was given intravenously, 1 g daily for 5 days, followed by prednisone

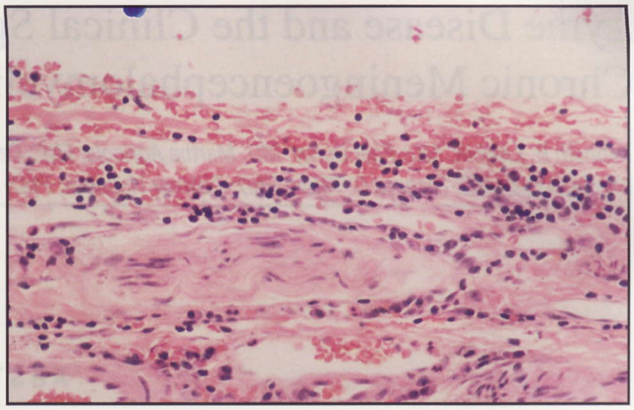

Fig 2, Case 1: Hematoxylin and eosin stain of pericardium removed when pericardial "window" was created, showing pericarditis with infiltration by plasma cells and macrophages [original magnification 400x].

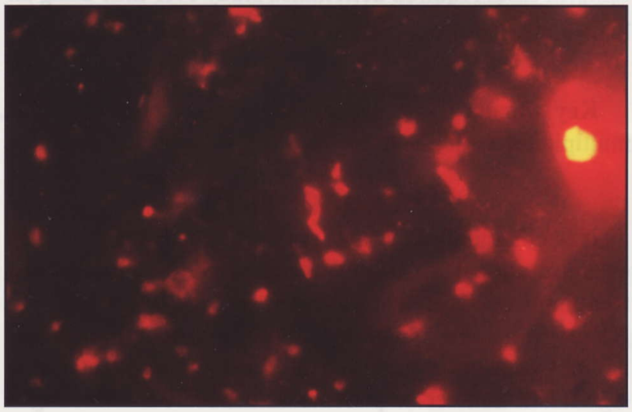

Fig 4, Case 1: Phycoerythrin stain of pericardial tissue demonstrating up-take by spirochetal-compatible form [original magnification 1000x].

over a six-week period for the possibility of systemic lupus erythematosus. Pleural effusions developed within one week of starting steroids along with severe encephalopathy and debilitation. She could not remember conversations held minutes earlier and was unable to hold a cup, roll over in bed, or transfer from bed to wheelchair. Computed axial tomography of the chest revealed pleuropericardial effusions (Fig 1).

A pleuropericardial window was created for diagnostic and therapeutic purposes. Fibrinous pericarditis was present with infiltration of plasma cells and macrophages and spirochete-compatible structures were seen with modified Steiner silver and phycoerythrin stains, as well as a touch preparation (Figs 2-5).

Intravenous CFOTX 6 g daily was administered for the next 3fi months with dramatic improvement of her encephalopathy. The pleuropericardial effusions improved (Fig 6). The patient was able to walk 500 feet

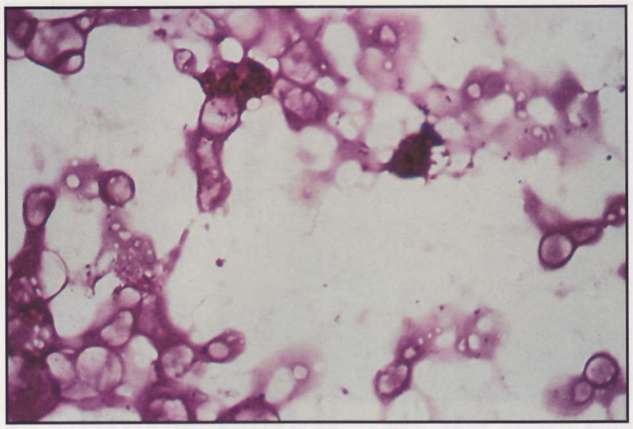

Fig 5, Case 1: Touch preparation of pericardium, showing spirochete-compatible structure [original magnification 1000x].

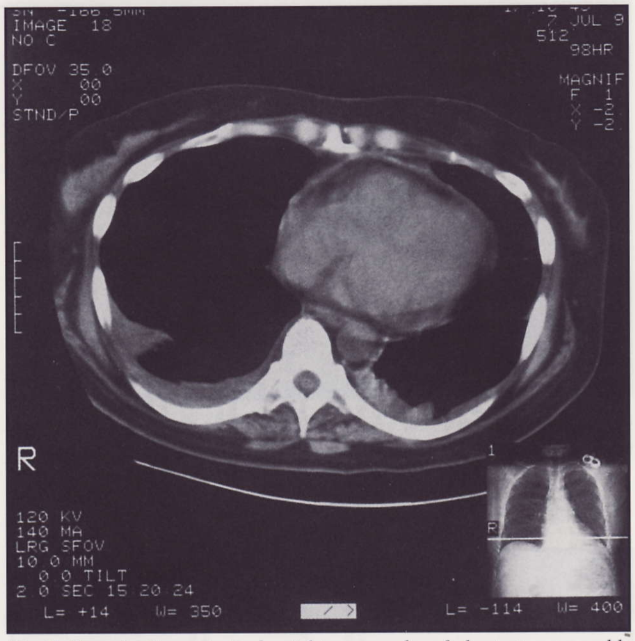

Fig 6, Case 1: Computerized axial tomography of chest at comparable level to Figure 1, after patient had received some two months of daily intravenous cefotaxime following creation of pericardial "window." Pericardial effusion and bilateral pleural effusions are significantly diminished.

with a rolling walker and was able to go home. A further 3 months of daily CFOTX was administered but the patient's health insurer refused authorization for any subsequent intravenous antibiotic therapy.

The patient became increasingly encephalopathic over the next 6 months. Daily intravenous CFOTX was reinstituted in June 1994 and mental status improved as confirmed by serial neuropsychological testing before and after 4 months of treatment.

Several specimens of plasma and urine between February and July of 1995 were found to be PCR positive for *B. burgdorferi*-specific DNA. From July 1995 through April 1996 the patient was treated with intramuscular benzathine penicillin. On this treatment she felt poorly, encephalopathy worsened, and she lost the ability to ambulate. Plasma PCR for *B burgdorferi*-specific DNA was again positive February 1996. CSF analysis March 1996 showed 14 lymphocytes/mm^3, elevated protein (57 mg %) and slight elevation of IgG. Oligoclonal bands were present in *both* CSF *and* serum. Myelin basic protein was absent. CSF Lyme PCR and OspA antigen were negative as were Lyme-specific immune complexes in serum and CSF. Authorization for additional intravenous antibiotic therapy was refused by the insurer. Encephalopathy and debilitation worsened (Table I).

Case #2

In the fall of 1985 a 61-year-old outdoorsman residing in the Catskill region of New York State developed a large round rash on one thigh. A physician was consulted but no treatment was given. The following winter unrelenting headache, low grade fever, paresthesias and truncal instability developed. Lumbar puncture demonstrated lymphocytic pleocytosis. Lyme ELISA was negative. Dysphasia and a progressive stroke syndrome developed. A diagnosis of "vasculitis" was given and the patient was treated with steroids and cyclophosphamide for a number of months with progressive deterioration to a level of functioning slightly above a persistent vegetative state. Lyme ELISA was positive in 1988. Treatment with intramuscular ceftriaxone (CFTRX) for 14 days resulted in slight improvement.

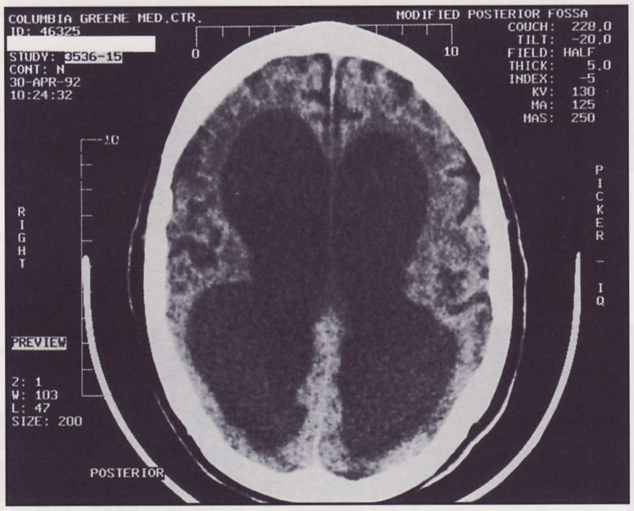

Fig 7, Case 2: Computerized axial tomography of the head showing massive hydrocephalus, May 1992, 6fi years after an untreated skin eruption historically compatible with erythema migrans.

In 1992, computed axial tomography of the brain

Table I (Case 1)

Clinical	Diagnostics	Treatment
1960-1970 Patient lives in Westchester County, NY in wooded setting with abundant deer		
1973-1976 Patient lives in Northern California; Extensive hiking, Marin County.		
1975 Idiopathic thrombocytopenic purpura develops.		
1976 Splenectomy.	1993 Paraffin sections of spleen neg. for spirochetes on silver staining (P.Duray); tissue PCR neg. (D. Persing).	
1977 Suffers "nervous breakdown", depression & suicidal ideation.		
Fall 1987, at age 37, gait disturbance develops.	1/88 CSF: 10 WBC/mm^3 protein 49 mg %	
1989 Spastic hemiparesis . diplopia, bilateral babinski absent gag, clonus, dysphonia, decomposition of movements noted.	1989 ANA 1/320 diffuse Anti-extractable nucl antigen AB neg. Anti-DS DNA AB neg. HIV neg. Lyme Elisa neg MS panel neg.<br>7/89 CSF:19 WBC/mm^3 86% lymphs 14% monos:	
	1/90 Raji cell elevated; IgG ACLA elevated; HTLV-1 neg.	
	1/18/90 CSF: 6 WBC/mm^3 93% Lymphs 5% polys prot NL. OCB neg. MBP neg. CSF IgG elev.<br>Paired CSF/serum Lyme Elisas neg.<br>CSF Lyme PCR neg.	1/18/90 CFOTX 2g IV Q 8hrs X 21days.
	2/7/90 CSF: WBC 9/mm^3 93% lymphs 7% mono	
	2/90 serial CSF exam X 4 all show pleocytosis.	
		3/90 trial of prednisone X 5weeks. No benefit.
	10/90 Lyme Elisa borderline. WB neg.	
		11/90-3/91 minocycline 200 mg./day. No benefit.
	12/90 T-cell stim. test positive.	
	12/91 CSF: WBC 6/mm3 89% lymphs 6% polys prot 61 mg%. CSF IgG increased<br>CSF IgG synth rate increased Paired serum/CSF Lyme Elisas + CSF/serum ratio 1.04<br>Serum WB: IgM 37,38,41,45, 64,72,83<br>IgG 18,22,23,28,30,35,62,93 CSF OspA antigen +	
	Bb grows in culture.<br>Gel electrophoresis oligonucleotide pattern shows "resemblence to West coast isolates" (D. Persing).	
	1/27/92 CSF: WBC 10/mm3 prot 44 mg%<br>BSK Bb cult X 4 aliquots all neg. Bb PCR +.	
		1/27/92 "pulse" CFOTX 4 g Q8 X 3 doses once weekly begun.
		4/92 "pulse" CFOTX changed to 4 g Q8 X 6 doses weekly
4/92 hypersomnolence and fatigue resolves. Less slurring of speech.		
	4/30/92 CSF: WBC 1/mm^3 prot 51 mg%. CSF IgG incr. IgG synth rate WNL paired Elisas + CSF/serum 1.18	
	7/92 CSF: WBC 1/mm^3 96% lymphs 4% monos	
9/92 gradual continued neurologic deterioration.	9/92 CSF: WBC 5/mm^3 CSF IgG incr.<br>paired Elisas + CSF/serum .98	
12/92 inappetance, increasing spasticity, patient transferred to tertiary care center for consideration of baclofen pump for spasticity.	12/92 CSF: WBC 5/mm^3 WB IgG: 39,41,60	12/92 "Pulse" CFOTX discontinued.
	CT scan abdomen: no pleural effusions seen	

Table I (Case 1)

Clinical	Diagnostics	Treatment
	12/92 CSF: WBC 8/mm3 78% lymphs 11% monos<br>Serum WB 25,30,41,42,59,60<br>ANA 1:2560 nucleolar	
		12/30/92-1/12/93 CFTRX 2 g/day
		1/21/93-1/25/93 Methylprednisolone Sodium Succinate 1 g/day
1/29/93 right pleural effusion noted; enlarges and becomes bilateral; increased vertical diplopia.	1/28/93 CSF: WBC 3/mm^3 81% lymph 7% monos<br>1/29/93 thoracentesis; pleural fluid thought compatible with "lupus serositis."<br>2/93 HIV neg.	2/3/93-3/1/93 prednisone 60 mg/d<br>3/2/93-3/7/93 prednisone40 mg/day
3/93 transferred back to referring community hospital without baclofen pump.	3/93 ESR > 100 mm/hr CSF: WBC 2/mm^3 92% lymph CSF IgG and IgG synth. rate WNL<br>OCB & MBP neg.<br>Paired Elisas CSF/serum .88	
	3/93 repeat thoracentesis: pleural fluid complement WNL; Bb PCR + in one lab, negative in another.	
5/93 develops severe encephalopathy. Unable to hold a cup, roll over in bed, or transfer from bed to wheelchair. Aspirating.		
	5/93 CT scan of chest: sizable bilateral pleural effusions and pericardial effusion.	
5/93 pleuropericardial window for diagnostic and therapeutic purposes.		
	Pericardial histology: fibrinous pericarditis infiltration of plasma cells and macrophages. Two separate spirochete compatible structures seen on silver staining. Phycoerythrin positive	
	5/93 CSF: WBC 5/mm^3 OspA PCR + in one lab, negative in another<br>6/93 skin biopsy "lupus band" test negative.	5/93-10/93 CFOTX 2g IV Q 8hr daily X 109 Continuous days.
5/93-7/93 gradual improvement in encephalopathy, gait, muscle strength.		
	7/93 CT chest shows significant decrease in pleural and pericardial effusions.	
9/93 able to walk 500' with a rolling walker.	ESR 40-60 mm/hr	
10/93 discharged home with home health aide; greatly improved.		
1/94 relatively stable neurologically; able to function at home with support. Insurer refuses reimbursement for further intravenous antibiotic therapy.		
1/94-6/94 gradually increasing spasticity, encephalopathy recurs and worsens, hypersomnolence, inappetance and nausea develop.	3/94 WB negative<br>ANA 1:1280 nucleolar	
	6/94 CSF: WBC 0/mm3<br>Paired Elisa + CSF/serum 1.01 CSF IgG incr.<br>Serum WB 41,60,64	
		10/94-2/95 CFOTX 2 g Q8 daily
11/94 independent in many ADL; nausea and hypersomnolence resolves.		
	2/95 neuropsychological testing documents modest improvement in cognitive function after 4 months of daily IVAB Rx.<br>2/95 Bb PCR + plasma<br>4/95 Bb PCR + plasma<br>7/95 Bb PCR + urine; HGE AB negative.	4/95-7/95 CFOTX2 g Q hr IV daily Rx
7/95 IVAB Rx suspended due to lapse in insurance coverage.		8/95-4/96 Benzathine PCN 1.2 MU weekly.
	2/96 Lyme Elisa + 1:320 WB 18,32,39,60,62	
Spring 96 worsening encephalopathy, fatigue, arthralgia, myalgia, "sick" feeling.		
	3/96 CSF: WBC 14/mm3 prot 57 mg% OCB present in CSF and serum MBP neg CSF IgG slightly incr. IgG synthesis rate WNL paired Elisa +CSF/serum 1.08 WB serum IgM 23,60,64,68<br>IgG 23,28,41,43,45,58,60,62,64 Bb PCR urine, plasma, CSF all neg OspA antigen and Lyme-specific immune complexes negative in CSF; + IgM Lyme-specific immune complexes in serum	Plan to seek authorization for reimbursement for reinsitution of open-ended IVAB Rx

Note: Please see abbreviations and reference ranges for Tables I-IV following Table IV.

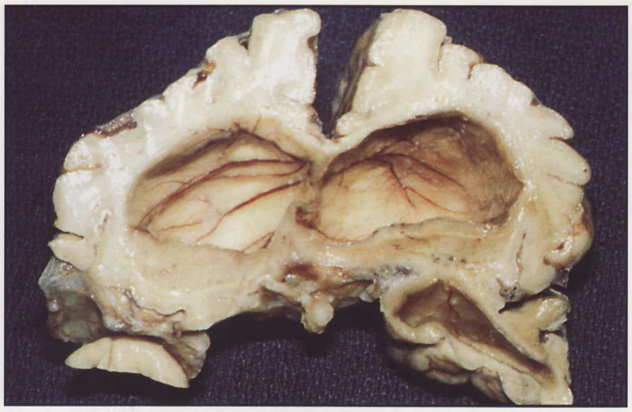

Fig 8, Case 2: Coronal section of brain at level of the temporal horns showing massive hydrocephalus at autopsy, July 1993.

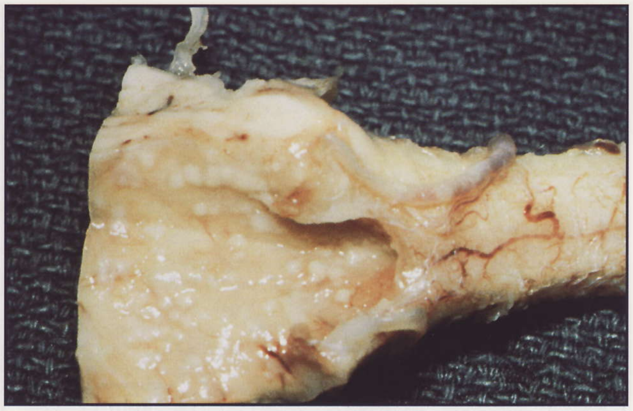

Fig 9, Case 2: Floor of the fourth ventricle and brainstem viewed following removal of cerebellum, at autopsy, July 1993. Prominent ependymitis is evident.

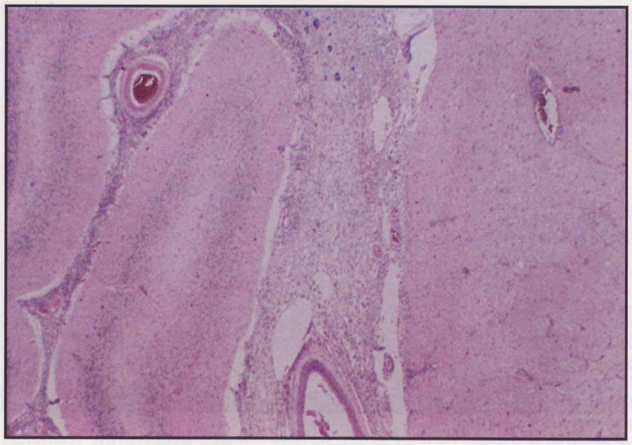

Fig 10, Case 2: Section through cerebellar cortex overlying floor of the fourth ventricle at the foramen of Luschka, showing florid granulomatous meningoencephalitis and ependymitis [original magnification 40×].

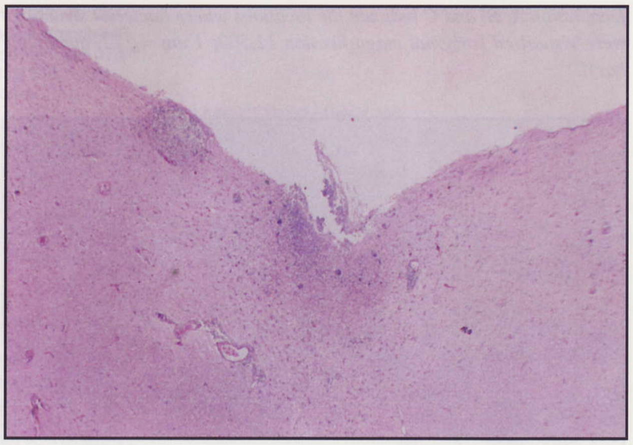

Fig 11, Case 2: Floor of the IVth ventricle showing mixed granulomatous and acute inflammation [original magnification 40×].

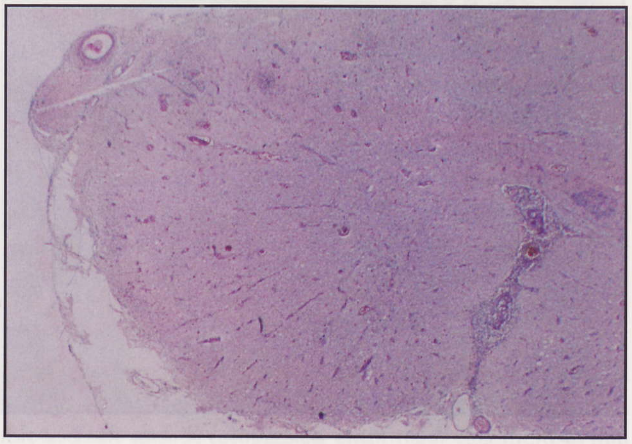

Fig 12, Case 2: Anterior quadrant of thoracic spinal cord showing meningitis and focal myelitis [original magnification 40×].

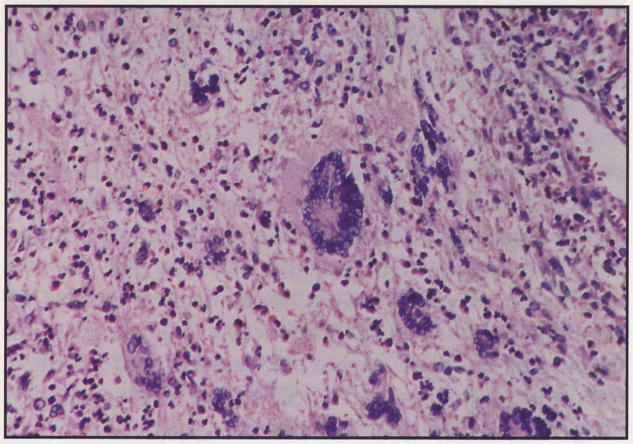

Fig 13, Case 2: Higher power view of the foramen of Luschka granulomatous inflammation showing giant cells, mononuclear cells, and polymorphonuclear leukocytes [original magnification 400×].

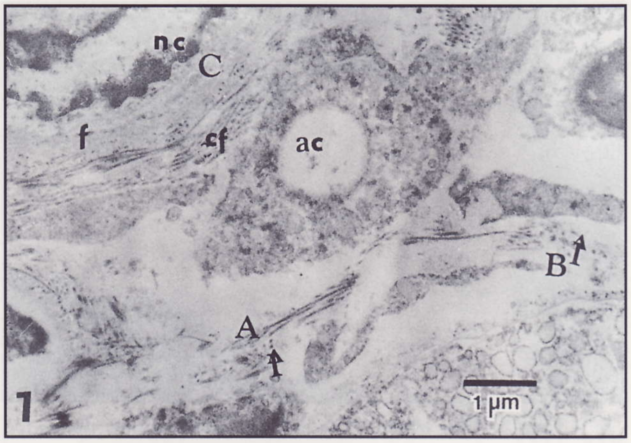

Fig 14-1, Case 2: Transmission electron microscopy of brain tissue from a formaldehyde-fixed autopsy. This cross section shows localization of dense bacteria (arrows) in collagen fibers (cf) and in fibroblast (f) near an altered capillary (ac). nc indicates the nucleus of the fibroblast. Denotation A, B, and C indicate the locations where bacterial structures were visualized [original magnification 12,500; 1 um = 12.5 mm (scale bar)].

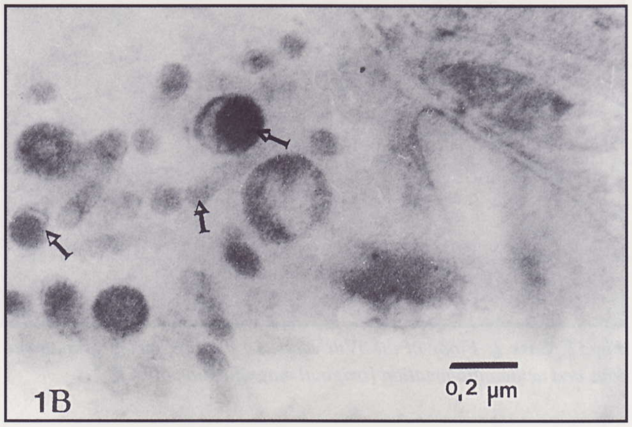

Fig 15-1B, Case 2: High magnification insert from Fig. 14, location B shows cluster of bacteria (arrows) cut in varying diameters. Some sections are from the thin longitudinal ends and some are from central 0.22 micron thick dense region of the spirochetes (arrow) [original magnification 60,400x, uranyl acetate, lead citrate].

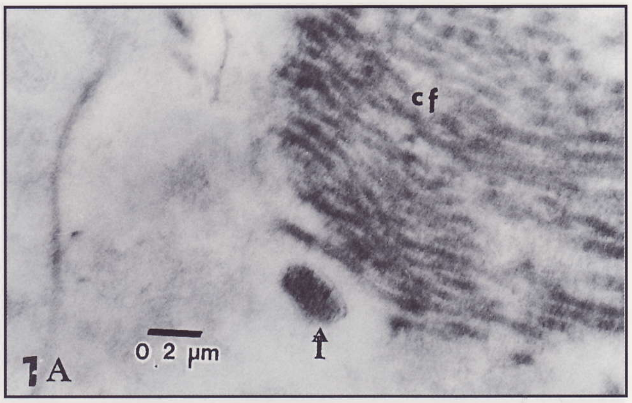

Fig 15-1A, Case 2: High magnification insert from Fig 14-1, location A. Arrow points to cross section of spirochetal bacteria-compatible structure with dense ribosomes surrounded by a surface membrane near the collagen fibers (cf) [original magnification 46,400x, uranyl acetate, lead citrate].

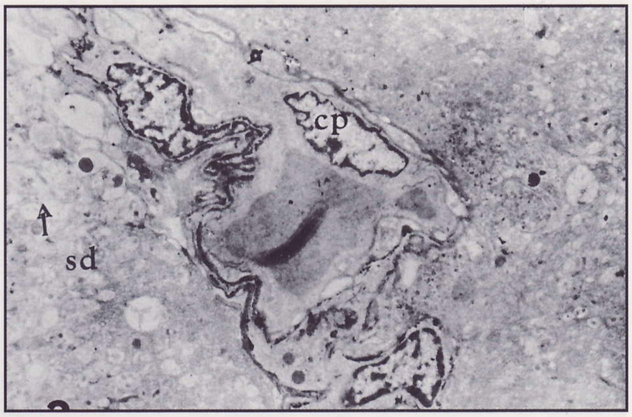

Fig 16-1. Case 2: View of brain section through axodentritic terminals near a blood vessel surrounded by pericytes (cp) [original magnification 3900x, formaldehyde-fixed tissues]. At the synapse (sd) the intracellular gap is increased (arrow) and there is dense extracellular material applied to the cytoplasmic side from which insert is made.

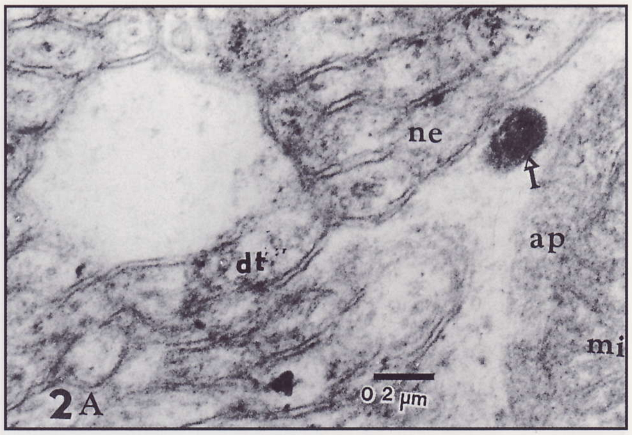

Fig 16-1A, Case 2: Insert shows some sections of dense ribosome-rich bacteria (arrow) surrounded by a neuroglial process (ap) with mitochondria (m) and by dendritic terminals (dt) [original magnification 52,600x, uranyl acetate, lead citrate]

showed massive hydrocephalus (Fig 7). Electroencephalogram revealed status epilepticus and phenobarbital was prescribed. Lyme serology was negative in one laboratory, yet positive in another. Western blot was nondiagnostic, showing only a 41 kiloDalton band. CSF examination revealed the presence of oligoclonal bands without myelin basic protein and very elevated CSF IgG. Serum showed elevated C1Q immune complexes. OspA antigen capture assay in CSF was strongly positive.

The patient was given daily intravenous CFTRX for one month, then weekly CFOTX (4 g IV Q 8 hr x 3 doses) for one year, with modest improvement in his neurologic status. The patient succumbed to his disease July 1993.

Table II (Case 2)

Clinical	Diagnostics	Treatment
10/85 61 year old Catskill region outdoorsman develops eruption compatible with erythema migrans one thigh.		
1/86 Develops unrelenting headaches,low grade fever, paresthesias, truncal instability. Evolves to progressive stroke syndrome. Diagnosis of "vasculitis" made.	1/86 CSF lymphocytic pleocytosis. Lyme Elisa negative.	
		Winter-Spring1986 prednisone and cytoxan given
Progressive neurologic deterioration		
	1988 Lyme Elisa +	
		CFTRX 2 g/day IM X 14 days
Slight improvement noted.		
1988-1992 patient cared for at home. Exists at primitive level of neurologic function; dependent on others for total care.		
5/92 status epilepticus; primitive emotive vocalization; slightly above vegetative state.		
	5/92 CSF: WBC WNL CSF IgG 17.2 mg/Dl CSF IgG synthesis rate 43.4 mg/24 hr. OCB + CSF MBP neg. CSF OspA antigen + 0.12/.04 CSF Lyme specific immune complexes strongly +(P. Coyle); serum C1Q immune complexes + 37.5 mcg AHG Eq/M ACLA IgG 25.9 GPL Lyme Elisa seroequivocal. WB negative.	
		5/92-6/92 CFTRX 2 g/day X 28d 7/92-6/93"pulse" CFOTX 4 g IV Q 8 X 3 consec. doses weekly.
7/92-6/93 modest neurologic improvement corroborated by visiting nurses.		
	2/93 Lyme Elisa .150/.107 WB negative C1Q immune complexes 54.4 mcg AHG Eq/M	
7/93 patient dies.		
	Autopsy: fulminant meningoencephalomyelitis and ependymitis; CSF OspA antigen + .087/.074 Lyme-specific immune complexes IgG + .944/.053 (P. Coyle); Silver staining and immunohistochemistry fails to reveal any definite spirochete-compatible structures (P. Duray, M. Philipp). Bb PCR positive widely in CNS tissues; Electron microscopy reveals borrelia- compatible structures (D. Hulinska)	

Autopsy revealed severe hydrocephalus (Figs 8,9) and florid meningoencephalomyelitis and ependymitis (Figs 10-13). The CSF was positive for OspA antigen and Lyme-specific immune complexes. Spirochetes were not visualized on histopathologic and immunohistochemical study by light microscopy but borrelia-compatible structures were visualized in formalin-fixed tissues studied by electron microscopy (Figs 14-16) and brain tissue and dura mater were PCR positive for detection of *B. burgdorferi*-specific oligonucleotides (Figs 17A,B)[7] (Table II).

Case 3

A 37-year-old woman removed a tiny tick from her left shin in the spring of 1982 while visiting Dutchess County, New York and developed an eruption about the site that persisted for several years. Biopsy of the lesion was read as granuloma annulare. Multisystem symptomatology developed within months of the attachment including polyarthralgia and synovitis, fatigue, headache, paresthesias, cognitive problems, and ocular disorders incuding pars planitis, anterior and posterior granulomatous uveitis, and retinal vasculitis.

Several Lyme ELISAs between 1982 and 1990 were negative. A short course of doxycycline in 1990 conferred some benefit. In June 1990 intravenous CFTRX 2 g/day was given for 42 days with symptomatic improvement. She was then given minocycline, 300 mg/day, for the next 2fi years with progressive improvement.

In the spring of 1993 the patient used minocycline sporadically, and in the summer of 1993 she developed neurologic symptoms. MRI of the cervical spinal cord showed high intensity lesions (Fig 18). Cerebrospinal fluid examination revealed 70 cells (mostly lymphocytes) and markedly elevated protein and IgG. Oligoclonal bands were present in CSF but myelin basic protein was within normal limits. Lyme serologic tests were negative

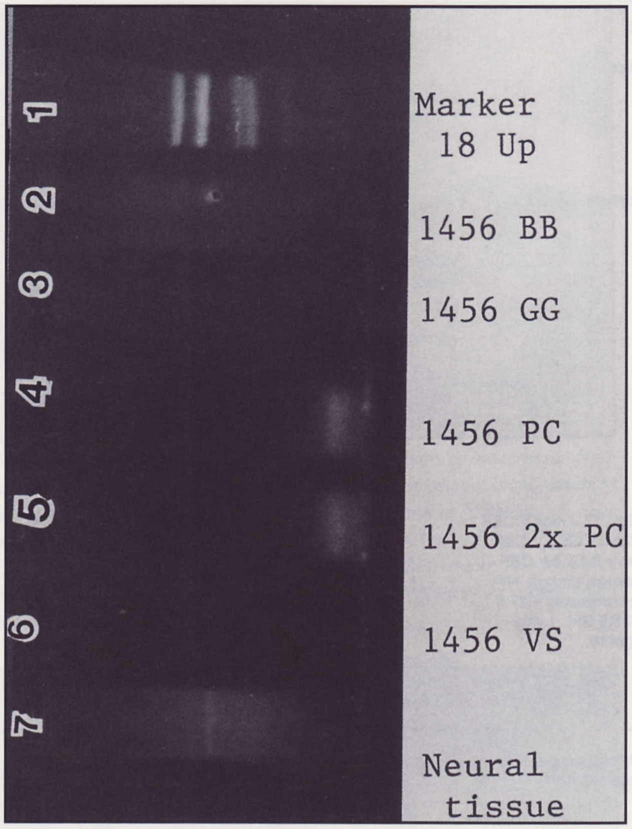

Fig 17-A, Case 2: PCR detection of B. burgdorferi *in brain autopsy tissues. DNA mass was isolated by the DNA QIA Amp Tissue kit QIAGEN (Boehringer Manheim) and subjected to analysis by PCR amplification using primers BB, GG, PC, VS. The reaction products were electrophoresed in 1.5% agarose gel and detected by UV transillumination after being stained with ethidium bromide. Lanes 2,3,6 did not contain PCR products with European primer sets BB, BG (for* B. garinii*), VS (for* B. afzelii*). Lane 1 contains DNA marker pUC18 Hae III digest (Sigma), lanes 4 & 5 contain PCR products amplified with primer set PC from a North American isolate.*

in serum and CSF as was CSF culture for *B. burgdorferi,* and OspA antigen in CSF, and PCR and Lyme-specific immune complexes in CSF and serum.

The patient was treated with daily intravenous CFOTX for the next 8 months during which time serial CSF examinations revealed progressive decrease in pleocytosis, protein, IgG and IgG synthesis rates. The last 3 of 5 CSF samplings showed Lyme-specific immune complexes in CSF and Lyme Western blots now demonstrated key *B burgdorferi*-specific bands (IgG: 28, 30, 39, 93 from one lab and 23, 29, 39 from a second lab). Intravenous antibiotics were discontinued July 1994.

The patient has been maintained on weekly intramuscular benzathine penicillin (4.8 million units IM), combined with azithromycin (750 mg/day) since that time, remaining clinically well. Western blots from February 1996 showed the 39 kDa band on IgM blot and 28, 30, 39,

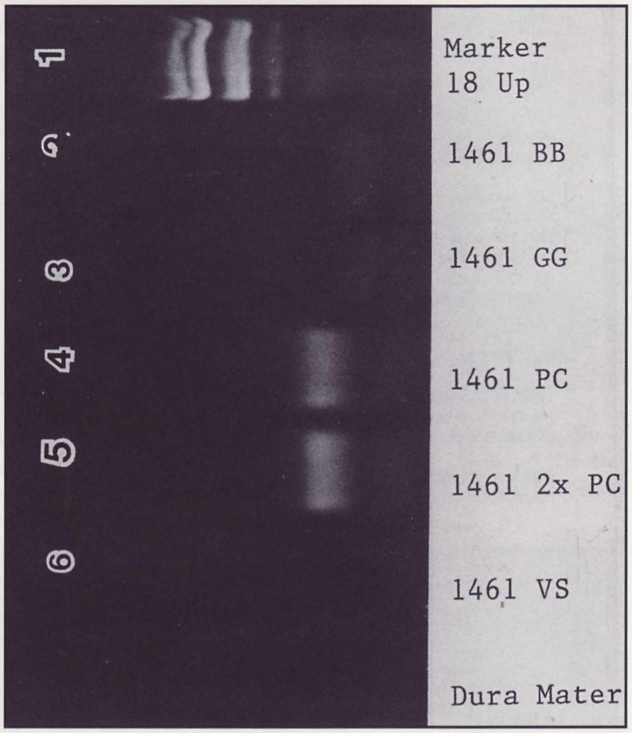

Fig 17-B, Case 2: PCR detection of B. burgdorferi *DNA in dura mater using primers BB, GG, PC, VS, and Fla I,II. Lanes 2, 3, & 6 did not contain PCR product. Lanes 4 & 5 contain PCR product with primer set PC (fragment of 102 base-pairs and lane 7 contain a specific fragment of 372 base pairs).*

41 kiloDalton bands on IgG blot and July 1996 showed 20 and 34 kDa bands on IgM and 28, 34, 39, and 58 kDa bands on IgG. Lyme ELISA has been negative throughout (Table III).

Case 4

In October 1989 a 40-year-old fire captain developed optic neuritis, constitutional symptoms, and progressive neurologic symptomatology believed on clinical grounds to be multiple sclerosis. MRI of the neuraxis showed hyperintense lesions at a variety of levels. CSF examination was not performed. His condition progressively deteriorated to a wheelchair-bound status despite treatment for multiple sclerosis, including beta interferon. There was no history of deer tick attachments nor of erythema migrans but he had had a large-type tick attachment occurring in the 1970s on Parris Island, South Carolina, followed by some joint symptoms. He had spent a great deal of time in shore areas of Rhode Island, Connecticut, and Massachusetts. All Lyme serologic tests and research assays in blood and CSF were negative in September 1994 and CSF showed a pattern thought pathognomonic for multiple sclerosis.

In view of the patient's lack of response to treatment

Table III (Case 3)

Clinical	Diagnostics	Treatment
5/82 37 year old woman removes an embedded tiny tick from the left shin after visiting rural Dutchess County, NY. 6" diameter ring-like rash develops, centered about the bite site. This faded slowly but persisted until 1987.		
7/82 Knees become reddened and swollen.		
1/83 Severe flu-like illness lasting three weeks occurs, with temperature as high as 104 degrees Farenheit.		
	1986 Residua of rash on shin biopsied: "granuloma annulare" Silver staining performed on paraffin block 11/94: "no spirochetes visualized".(P. Duray)	
August 1982-1990 Ocular problems develop: anterior & posterior granulomatous uveitis, pars planitis, retinal vasculitis with pre-retinal fibrosis and bilateral cataracts.		Topical indocin and oral and topical steroid Rx for eyes.
	1988 Lyme Elisa neg<br>1/90 Lyme Elisa neg.	3/90 oral doxycycline for 21 days.
1989-1990 Paresthesias occur involving upper extremities and fatigue.		
	5/90 Lyme Elisa neg.	
	6/90 Lyme Elisa neg.	6/90-8/90 CFTRX 2 g/day IV for 6 weeks.
8/90 Clear improvement in ocular findings and arthralgias and paresthesias resolve.	7/90 ANA neg	
		8/90 Minocycline begun, 100 mg Q 12 hr<br>10/90 Minocycline increased to150 mg Q12 hr. Remains on this dose for about 2 years.
10/90 Feeling well.		
11/90 Eyes stable; no new lesions.		
6/91 Vision progressively improving.		1992 Patient tapers minocycline to 200 mg/day on own and is sporadic about dosing.
6/93 Patient seen after lapse of 14 months.	6/93 Lyme Elisa neg.	
9/93 Numbness develops in both hands, balance off, Lhermitte's sign, hip pain.	9/93 MRI Cervical spine shows hyper-intense lesions at C2, C4, C6-7. MRI of brain WNL.	9/93 minocycline suspended.
	11/93 CSF: WBC 70/mm3 90% lymphs 9% monos 1% plasma cells protein 87 mg% (ULN 45 mg%) paired Lyme Elisas neg. CSF IgG 33 mg/Dl IgG synthesis rate 140.5 mg/24 hr. OCB + (serum neg) MBP WNL CSF OspA antigen neg.; Serum and CSF Lyme-specific immune complexes neg. Bb culture neg Bb PCR neg serum, urine, CSF; Viral culture neg. CSF; cytology: reactive lymphocytes with plasmacytoid changes.	
11/93 Fatigue hip pain, right arm numbness increase two weeks into IVAB Rx.		11/93 IV CFOTX 2 g Q 8 hr. begun.
12/93 Balance improved and Lhermitte's diminished 6 weeks into IVAB Rx.		
1/94 Balance now near normal, fatigue almost resolved, diminished paresthesias of torso and LLE after 8 weeks IVAB Rx.		
1/94 Dizzyness completely resolved, numbness in hands only involves finger tips and toes after 11 weeks IVAB Rx.		
2/7/94 Arthralgias essentially completely resolved.		
	2/94 T-cell stimulation test negative. 3/94 CSF: WBC 14/mm3 97% lymphs protein 57 mg%	

Table III (Case 3)

Clinical	Diagnostics	Treatment
	CSF IgG 29.2 mg/Dl IgG synthesis rate 121 mg/24 hr OCB + (serum neg) MBP WNL viral culture neg.; paired serum/CSF Elisas neg. CSF OspA antigen neg.; CSF and serum Lyme specific immune complexes neg.	
3/94 Hips develop dull ache within 2 weeks of adding azithromycin.		3/94 CFOTX dose increased to 8 grams/day azithromycin 500 mg/day added
4/94 Balance normal, feet and legs now normal sensation, arthralgias absent, Lhermitte's very mild. Good energy. No longer requires day-time naps.	4/94 WB serum, neg.	
	5/94 CSF: WBC 9/mm3 95% lymphs protein 42 mg% OCB + (serum neg) MBP WNL CSF IgG 19.3 mg/Dl. IgG synthesis rate 81.6 mg/24 hr. paired Lyme Elisas neg paired WBs neg CSF OspA antigen neg. serum Lyme-specific immune complexes neg CSF Lyme-specific IgG immune complexes + .175/.123 IgM neg.	
6/94 All leg numbness resolved following 7 months IVAB Rx.		
	7/94 CSF: WBC 7/mm3 99% lymphs prot 42 mg% OCB + (serum neg) MBP WNL CSF IgG 14.3 mg/Dl IgG synth rate 55.4 mg/24 hr paired Lyme Elisas neg serum Lyme-specific immune complexes neg. CSF OspA antigen neg. CSF IgG Lyme-specific immune complexes +.170/.123 IgM neg.	
7/94 Medically and neurologically stable.		7/94 IVAB Rx stopped after 8 months of treatment. Benzathine PCN 2.4 MU IM weekly begun; azithromycin continues. 8/94 Benzathine PCN increasedto 4.8 MU IM weekly azithromycin increased to 750 mg/day.
10/94 Essentially normal neurologic exam and status except for palm numbness related to carpal tunnel syndrome.		
	11/94 WB 30 kDa band.	
12/94 Vision showing continued improvement.Constitutionally well.Ocassional mild Lhermitte's. Stamina good. Joints pain free.		
	2/95 CSF: Bloody tap WBC, prot, CSF IgG and IgG synth rate uninterpretable. paired Lyme Elisas neg. WB serum IgG 28,30,39,93<br>2nd WB IgG 23,29,39 serum Lyme-specific immune complexes neg.; CSF OspA antigen neg.; CSF Lyme-specific IgG immune complexes + 296/.123 IgM immune complexes +.437/.041<br>Bb PCR urine, plasma, CSF neg.	
		3/95 Minocycline added for triple anti- biotic Rx with azithromycin and benzathine PCN.
4/95 Fully functioning; occasional hip arthralgias.		
	5/95 Lyme Elisa neg WB IgG 39,60,72 2nd WB IgG 39 kDa only	
9/95 Medically and neurologically stable.		
11/95 Stable.		
	1/96 WB 23,39 Bb PCR urine, plasma neg.<br>2/96 WB IgM 39 IgG 28,30,39,41,43,60,64	
		4/96 Remains on benzathine PCN azithromycin minocycline. Patient and physician fear discontinuing treatment.

Table IV (Case 4)

Clinical	Diagnostics	Treatment
1970's Tick removed while Marine at Parris Island, South Carolina.		
1980's Summers on coastal Connecticut island abounding in deer; also hikes on Block Island and dune areas Watch Hill, Rhode Island.		
8/87 6-7 months of tenosynovitis base of one thumb; also knee pain.		
10/89 Right optic neuritis.	1989 Lyme Elisa neg.	
1/90 Right optic neuritis.	1990 MRI of brain, high intensity lesions corona radiata	
3/90 Back pain.<br>8/90 Knee pain.	MRI LS spine L4-5 disc protrusions	L4-5 discectomy HNP
12/91 Fatigue, shoulder pain.		
1/92 Presumptive M.S. diagnosis; Winter-Spring needs cane to walk.		1/92 IV & IM ACTH no benefit<br>Spring '92 prednisone given; no benefit
12/92 Leg weakness, cervicalgia, depression.<br>2/93 Ceases work.		
9/93 Worsening neurologic status.		
12/93 Dysarthric, nystagmus, hyperactive reflexes, spasticity, dysmetria, scanning speech, using wheelchair, requires walker, walks with great difficulty. Gait lurching. Babinskis +.Knife-clasp spasticity lower extremities.		12/93 Beta-Interferon: no benefit.
	9/94 MRI brain multiple high intensity lesions deep white matter periventricular adjacent body of lateral ventricle and temporal horn; mild cerebral, cerebellar, pons, and mid-brain atrophy. 9/94 CSF: WBC 3/mm^3 protein WNL +OCB (serum neg) +MBP CSF IgG 6.5 mg/Dl. CSF IgG synthesis rate 12.8 mg/24 hr VDRL neg. Paired Lyme Elisas & WBs neg; Bb PCR neg; serum, urine, CSF; Bb CSF culture neg; OspA antigen in CSF neg; Lyme-specific immune complexes neg in CSF and serum.	
10/94 Synovitis ankles noted; knee aching.		10/94-5/95 empiric IVAB Rx CFOTX 2 g Q 8 hr.
11/94 Synovitis overlying knuckles.		
	2/95 CSF: WBC 0/mm^3 prot WNL. OCB neg in both CSF & serum MBP neg CSF IgG 3.9 IgG synth rate 8.32 paired Lyme Elisas neg WB serum: IgM 43,60,66,93 IgG 62,66,94 OspA antigen CSF neg. IgG Lyme-specific immune complexes + serum, neg in CSF Bb PCR neg serum, urine, CSF.	
11/94-4/95 Improved cognition, mood and memory; improved ability to transfer and ambulate with and walker, eventually walking at least 100'.Limb movements and speech more fluid. Cyclic synovitis.	3/95 MRI Brain: diminished number and size of lesions in deep white matter.	
	3/95 WB serum:IgM 30,45,58,93 IgG 41,64,66,80<br>4/95 WB serum:IgM 41,43,60,68,93 IgG 39,43,45,58,60,61,62,64,68	
	6/95 WB serum IgM 66,93 IgG 39,64	6/95 Benzathine PCN 2.4 MU IM/week begun; azithromycin added, 500 mg/day.<br>7/95 Benzathine PCN increased to 4.8 MU/week.
5/95-9/95 Clinically deteriorates markedly; loses ability to ambulate and develops paralysis of legs.Loses bowel and bladder control.	8/95 MRI of LS spine: no mechanical cause for neurologic impairment; no new cord lesions seen.<br>9/95 CSF: WBC 11/mm3 prot 63<br>OCB + CSF, neg serum MBP 10.2 mcg/ml CSF IgG 8.4 mg/Dl. IgG synthesis rate 20.6 mg/24 hr. Paired Elisas neg<br>WB serum IgM 60 IgG 39,60	
		9/95 CFOTX 2 g Q 8 IV begun.<br>11/95 CFOTX discontinued, imipenem/cilastatin begun 500 mg IV Q 8 hr.
9/95-3/96 Neurologic status improves; eventually can walk several hundred feet with a walker; regains bowel and bladder control; increased strength; diminished irritability; mood, cognition improved.		
	3/96 CSF: WBC 0/mm^3 OCB + in CSF and serum; MBP WNL CSF IgG 6.7 g/Dl IgG synthesis rate 16 mg/24 hr Bb PCR neg urine, plasma, CSF Bb culture neg.<br>WB serum: IgM 60 IgG 39,60,62 CSF: no significant bands OspA antigen CSF neg. Lyme-specific immune complexes in CSF and serum pending.	
		4/96 Imipenem/cilastatin dose increased to 1 g Q 8; anticipate need for open-ended IVAB

Abbreviations and Normal Reference Ranges (in parentheses) for Tables I-IV

ACLA: anticardiolipin, antibodies (IgG less than 23 GPL; IgM less than 11 MPL)

ANA: antinuclear antbodies

Anti-DS DNA AB: anti-double stranded desoxyribonucleic acid antibody

C1Q immune complexes: (less than 30 micrograms AHG Eq/mL)

CSF: cerebrospinal fluid

CSF IgG (0.70-3.50 mg/dL)

CSF IgG synthesis rate (0-10 mg/24 hours

CSF prot: CSF protein (20-45 mg/dL)

HIV: Human immunodeficiency virus

HTLV-1: Human T-cell lymphotropic virus

MBP: myelin basic protein (0-5.0 micrograms/L)

OCB: Oligoclonal bands (absent)

OspA: Outer surface protein A

Raji Cell assay: (0-50 mag AHG Eq/m)

aimed at multiple sclerosis, a five-month empirical treatment trial of daily CFOTX was given between the fall of 1994 and spring of 1995. The patient's neurologic status which had been progressively deteriorating, reversed. Wheelchair bound to start, the patient was able to ambulate at least 100 feet with a walker. Speech became clearer, and movements somewhat more fluid. Synovitis of ankles and knuckle joints was noted during the early phases of treatment.

MRI of neuraxis at completion of therapy showed improvement compared to a pretreatment study with a diminution in the number and size of lesions. CSF examination repeated at completion of 4 months of therapy showed disappearance of oligoclonal bands and myelin basic protein, normalization of IgG synthesis rate and CSF IgG only slightly elevated. Lyme Western blot although not fully diagnostic, showed evolution of key *B. burgdorferi*-specific bands (IgM 30, 45, 58, 93 kDa, March 1995; and IgG 39, 45, 58, April 1995) and Lyme-specific immune complexes were seen in serum for the first time (optical density 0.634; positive cut-off, greater than 0.215). OspA antigen in CSF remained negative.

While being treated with intramuscular benzathine penicillin combined with azithromycin, the patient deteriorated with loss of ability to ambulate and development of urinary and fecal incontinence.

Lumbar puncture repeated in September 1995 five months after discontinuing intravenous antibiotic therapy again showed markedly abnormal parameters indicative of multiple sclerosis including oligoclonal bands and elevated myelin basic protein, markedly elevated CSF IgG and IgG synthesis rate as well as lymphocytic meningitis.

Intravenous antibiotics were resumed. The patient regained his ability to ambulate with a walker and to control bowel and bladder. Repeat lumbar puncture March 1996 showed significant improvement in CSF parameters with disappearance of myelin basic protein, clearance of CSF pleocytosis, and decreased CSF IgG and IgG synthesis rate. Lyme-specific IgM immune complexes were detected in CSF (Table IV).

DISCUSSION

Seronegativity implies failure of detection of infection by the patient's immune defense and also masks the infection from recognition by the patient's physician. This may set the stage for the development of more serious neurologic or other systemic illness. Dattwyler et al showed that early application of antibiotic therapy may blunt the development of an antibody response.[8] Schutzer has shown that free antibodies may not be demonstrated unless methods to dissociate circulating immune complexes are used.[9] T-cell anergy may be another mechanism to explain both seronegativity and chronic persistent infection.[10] Recently, it has been found in *in vitro* experiments that *B. burgdorferi* may target, invade, and destroy human B- and T-lymphocytes and may even steal lymphocyte cell membrane.[11]

Analagous to the situation in leprosy,[12] there may be two clinical subsets of patients with Lyme disease defined by the host immune response. Both T- and B-cell deficiencies occurring in the seronegative subset may predispose to expression of more serious illness, as with lepromatous leprosy, and chronic persistent infection. The seropositive subset may resemble tuberculoid leprosy where the severity of illness is limited by the more effective host imune response. Other mechanisms, which may explain survival of borreliae despite antibiotic treatment include intracellularity[13,14] and the adoption of spheroplast-L forms to evade the lethal effect of cell wall-acting antibiotics.[15]

Case 1 showed clinical illness for at least four years prior to proof of diagnosis. Compelling laboratory evidence for chronic persistent infection was developed numerous times throughout her course including culture, demonstration of spirochetal-compatible forms in pericardial biopsy, and repeated PCR positivity all despite prior and sometimes very prolonged intravenous antibiotics. Weekly treatment with CFOTX was apparently not adequate to control the infection in her case. Maximal improvement was seen only after 6 months of daily intravenous antibiotic therapy, but the patient had suffered a degree of irreversible neurologic injury, and had relapses whenever treatment was discontinued.

This patient showed many markers suggesting lupus including positive ANAs, anticardiolipin antibodies, circulating immune complexes, and at one point even the presence of anti-double stranded DNA antibodies. Whether she suffered from a pre-existing connective tissue disease or the autoimmunity she evidenced was merely an epiphenomenon associated with chronic borrelial infection is unclear. However, treatment for systemic lupus erythematosus with corticosteroids resulted in severe clinical deterioration, whereas intensive antibiotic treatment reversed this situation and achieved dramatic improvement.

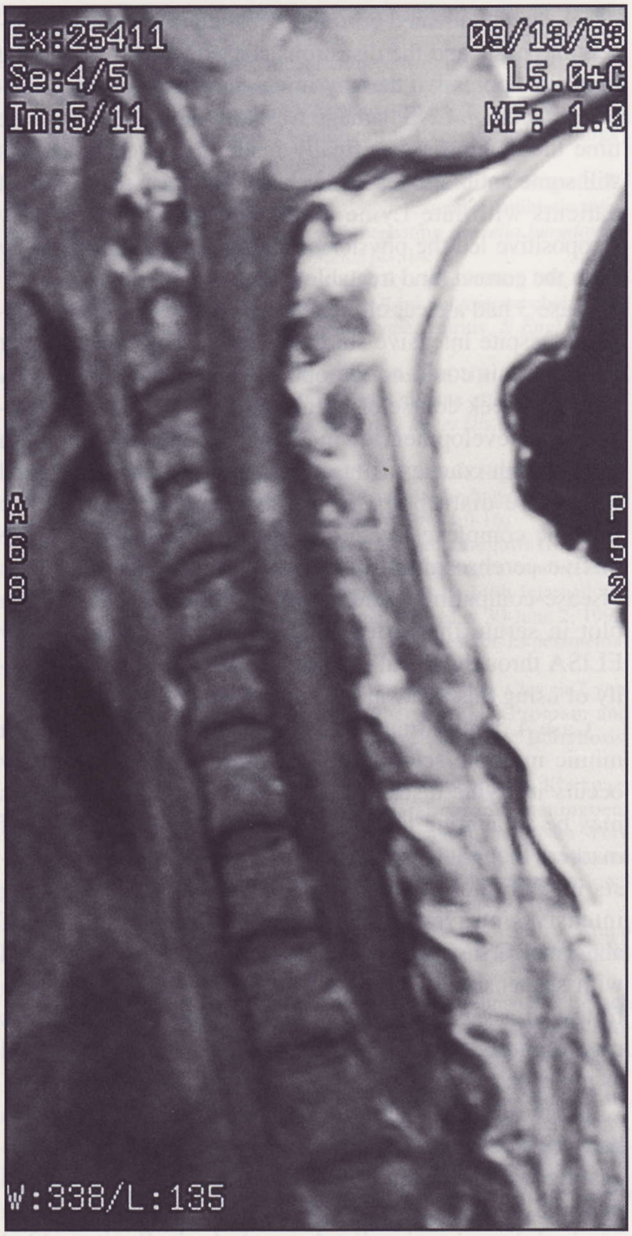

Fig 18, Case 3: MRI of cervical spine showing high intensity lesion within the cord in Case 3.

That her borrelial isolate showed resemblance to strains from the West Coast of the United States on pulse-gel electrophoresis, and that she had lived in California for a few years prior to the development of her ITP makes one wonder whether borrelial infection may have caused her ITP. One attempt to identify *B. burgdorferi*-specific DNA in tissue in paraffin blocks from the patient's splenectomy was negative, however. Her case prompts speculation on the possible role of borrelial infection in other autoimmune disorders previously thought to be idiopathic.[16,17]

Case 2 demonstrates the devastating potential of unrec-

ognized and untreated borrelial infection in genetically susceptible hosts and the disastrous consequences of applying immunosuppressive therapy in cases of unrecognized borrelial infection. CNS damage was very far advanced by the time the diagnosis was finally made; however, there was still some antibiotic responsiveness. The false teaching that patients with late Lyme disease are almost invariably seropositive led the physicians caring for this patient away from the correct (and treatable) diagnosis.

Case 3 had a clear clinical history indicating Lyme disease. Despite intensive study laboratory corroboration for the diagnosis could not be obtained for some 13 years. A prior six-week course of intravenous CFTRX did not prevent the development of meningoencephalomyelitis. An eight-month course of intravenous CFOTX was required to resolve disturbed CSF parameters. Lyme-specific immune complexes were demonstrable in the final three of five cerebrospinal fluid examinations and key Lyme disease-compatible bands finally developed on Western blot in serum thereafter. She has been seronegative by ELISA throughout, calling into serious question the validity of using this assay alone as a screening test.

Case 4 demonstrates how closely neuroborreliosis can mimic multiple sclerosis. Given now that seronegativity occurs in Lyme disease, distinguishing the two disorders may be a daunting task. The patient showed resolution of markers thought to be pathognomonic for multiple sclerosis in CSF along with clinical improvement following intensive intravenous antibiotic treatment. Relapse of abnormal CSF findings and of neurologic signs occurred with suspension of intensive treatment. Resolution again followed a second course of intravenous therapy. This case suggests that neuroborreliosis may be misdiagnosed as multiple sclerosis.[18,19]

On the other hand, a significant body of research data had been developed in pre-World War II Germany suggesting a relationship between spirochetal infection and multiple sclerosis.[20,21] Steiner averred that the *Spirocheta myelophthora* he visualized morphologically resembled borreliae rather than treponemes.[22] Additional studies using modern direct antigen detection techniques should be conducted to re-examine a potential relationship between borrelial infection and multiple sclerosis.

That borrelial infection may result in multiple sclerosis-like illness following months to years of clinical latency and without occurrence of erythema migrans ought to be taken into account in decisions on antibiotic prophylaxis for recognized ixodid tick attachments.[23-25]

CONCLUSION

Chronic persistent infection and seronegativity are not without precedent in spirochetal disese and are now well accepted phenomena in syphilis. Prolonged and combination antibiotic therapy are being utilized by an increasing number of clinicians to attempt to avert progressive neurosyphilis.[26] The hypothesis has been proposed that syphilis may not be a curable infection in the sense of total bacterial eradication with available treatment approaches.[27]

Relapses following use of potent antibiotics and detection of the Lyme organism or its DNA following treatment likewise demonstrates an inability to completely eradicate the pathogen and permanently halt the pathologic process with current methods of treatment in some patients.[28-47] This is a problematic situation because intensive antibiotic treatment is costly, is inconvenient, and carries associated risk for the patient.[48] Such antibiotic usage may foster the emergence of strains of other types of bacteria resistant to the antibiotics employed and thus has public health implications. For some patients, however, this may be the only presently available alternative to progressive neurologic deterioration.[38,39] In view of this dilemma, the international biomedical research community must give high priority to the development of improved and/or alternate methods of treatment that can definitively cure persisting borrelial infections responsible for neurologic and other manifestations of chronic Lyme disease.

ACKNOWLEDGMENTS

Patricia Coyle and Zhidian Deng, Department of Neurology, SUNY Stony Brook, performed OspA antigen and Lyme-specific immune complex testing in CSF and serum in Cases 1-4. The Centers for Disease Control and Prevention, Fort Collins, CO cultured for borreliae and did serologic and PCR testing in Cases 1 and 2. David Persing and Barbara Rutledge, Mayo Clinic Foundation, performed PCR testing and culture for borreliae in Cases 1 and 2 and pulse-gel electrophoresis characterization of the isolate in Case 1. Ben Luft, Department of Medicine, SUNY Stony Brook, provided the PCR primer used in studying autopsy tissues in Case 2. Mario Philipp and Dr. Roberts, Tulane Regional Primate Center, effected immunohistochemical study of CNS tissues in Case 2. Tom Gora provided computer expertise, hardware and software support and assisted in tabularizing data.

REFERENCES

1. Steere AC, Taylor E, McHugh GL, Logigian EL. The overdiagnosis of Lyme disease. *JAMA.* 1993;269:1812-1816.
2. Rahn DW, Malawista SE. Lyme disease: recommendations for diagnosis and treatment. *Ann Intern Med.* 1991;114:472-481.
3. Schoen RT, Malawista SE. Lyme arthritis. Cliniguide to rheumatology. Lawrence DellaCorte Publications, Inc. 1994;3:1-7.
4. Sigal LH. Current drug therapy recommendations for the treatment of Lyme disease. *Drugs.* 1992;43:683-699.
5. Gerber MA, Shapiro ED, Burke GS, Parcells VJ, Bell GL. Lyme disease in children in Southeastern Connecticut. *N Engl J Med.* 1996;335:1270-1274.
6. Duray P. Capturing spirochetes from humans [editorial]. *Am J Clin Pathol.* 1993;99:4-6.
7. Rosa P, Hogan D, Schwan T. Polymerase chain reaction analysis identifies two distinct classes of *B. burgdorferi*. *J Clin Microbiol.* 1991;29:524-532.
8. Dattwyler RJ, Volkman DJ, Luft BJ, Halperin JJ, Thomas J, Golightly MG. Seronegative Lyme disease. Dissociation of T- and B-lymphocyte responses to *Borrelia burgdorferi*. *N Engl J Med.*

1988;319:1441-1446.

9. Schutzer SE, Coyle PK, Belman AL, Golightly MG, Drulle J. Sequestration of antibody to *Borrelia burgdorferi* in immune complexes in seronegative Lyme disease. *Lancet.* 1990;335:312-315.

10. Schwartz RH. T cell anergy. *Sci Am.* August 1993:62-71.

11. Dorward DW, Fischer ER. Virulent *Borrelia burgdorferi* specifically attach to, activate, and kill TIB-215 Human B-lymphocytes [abstract]. VIII Annual Lyme Disease International Scientific Conference on Lyme Borreliosis and other Spirochetal and Tick-borne Diseases. Vancouver, BC. April 28,29, 1995.

12. Modlin RL, Bloom BR. Immune regulation: learning from leprosy. *Hosp Pract.* Nov 1993:71-84.

13. Georgilis K, Peacocke M, Klempner MS. Fibroblasts protect the lyme disease spirochete, *Borrelia burgdorferi*, from ceftriaxone in vitro. *J Infect Dis.* 1992;166:440-444.

14. Klempner MS, Noring R, Rogers RA. Invasion of human skin fibroblasts by the Lyme disease spirochete, *Borrelia burgdorferi. J Infect Dis.* 1993;167:1074-1081.

15. Preac Mursic V, Wanner G, Reinhardt S, Wilske B, Busch U, Marget W. Formation and cultivation of *Borrelia burgdorferi* spheroplast-L-form variants. *Infection.* 1996;24:218-226.

16. Lavoie PE, Kong L, Hogrefe W. *Borrelia burgdorferi* DNA in the blood of three SLE patients [abstract]. Program and abstracts of the Fifth International Conference on Lyme Borreliosis, Arlington, VA., May 30-June 2, 1992. Bethesda, MD: Federation of American Societies for Experimental Biology, 1992:A21.

17. Blaser MJ. Bacteria and diseases of unknown cause [editorial]. *Ann Intern Med* 1994;121:144-145.

18. Liegner KB. Evidence for borrelial etiology and pathogenesis in a series of patients carrying a diagnosis of multiple sclerosis [abstract]. 45th International Northwestern Conference on Diseases in Nature Communicable to Man. Vancouver, British Columbia, August 1992.

19. Liegner KB. Difficulty of distinction between borrelial (Lyme) encephalomyelitis and multiple sclerosis [abstract]. 47th International Northwestern Conference on Diseases in Nature Communicable to Man. Vancouver, British Columbia, August 1992.

20. Marshall V. Multiple sclerosis is a chronic central nervous system infection by a spirochetal agent. *Med Hypotheses.* 1988;25:89-92.

21. Kurtz SK. Relapsing fever/Lyme disease/multiple sclerosis. *Med Hypotheses.* 1986;21:335-343.

22. Steiner G. Acute plaques in multiple sclerosis, their pathogenetic significance and the role of spirochetes as etiological factor. *J Neuropathol Exp Neurol.* 1952;11:343.

23. Liegner KB. Lyme disease (letter). *N Engl J Med.* 1990;322:474-475.

24. Liegner KB. Prevention of Lyme disease after tick bites (letter). *N Engl J Med.* 1993;328:136-139.

25. Liegner KB. Antimicrobial prophylaxis after tick bites (letter). *N Engl J Med.* 1993;328:1418-1420.

26. Tramont EC. *Treponema pallidum (syphilis).* In: Mandell GL, Bennett JE, Dolin R (eds). Mandell, Douglas & Bennett's principles and practice of infectious diseases. Churchill Livingston. Fourth Edition: New York; 1995:2117-2133.

27. Ghinsberg RC, Nitzan Y. Is syphilis an incurable disease? *Med Hypotheses.* 1992;39:35-40.

28. Preac-Mursic V, Weber K, Pfister HW, Wilske B, Gross B, Baumann A, Prokop J. Survival of *Borrelia burgdorferi* in antibiotically treated patients with Lyme borreliosis. *Infection.* 1989;17:355-359.

29. Preac-Mursic V, Pfister HW, Spiegel H, Burk R, Wilske B, Reinhardt S, Bohmer R. First isolation of *Borrelia burgdorferi* from an iris biopsy. *J Clin Neuroophthalmol.* 1993;13:155-161.

30. Haupl T, Hahn G, Rittig M, Krause A, Schoerner C, Schonherr U, Kalden JR, Burmester GR. Persistence of *Borrelia burgdorferi* in ligamentous tissue from a patient with chronic Lyme borreliosis. *Arthritis Rheum.* 1993;36:1621-1626.

31. Lawrence C, Lipton RB, Lowy FD, Coyle PK. Seronegative chronic relapsing neuroborreliosis. *Eur Neurol.* 1995;35:113-117.

32. Liegner KB, Rosenkilde CE, Campbell GL, Quan TJ, Dennis DT. Culture-confirmed treatment failure of cefotaxime and minocycline in a case of Lyme meningoencephalomyelitis in the United States [abstract]. Program and abstracts of the Fifth International Conference on Lyme Borreliosis, Arlington, VA, May 30-June 2, 1992. Bethesda, MD: Federation of American Societies for Experimental Biology, 1992:A11.

33. Liegner KB, Shapiro JR, Ramsay D, Halperin AJ, Hogrefe W, Kong L. Recurrent erythema migrains despite extended antibiotic treatment with minocycline in a patient with persisting *Borrelia burgdorferi* infection. *J Am Acad Dermatol.* 1993;28:312-314.

34. Nocton JJ, Dressler F, Rutledge BJ, Rys PN, Persing DH, Steere AC. Detection of *Borrelia burgdorferi* DNA by polymerase chain reaction in synovial fluid from patients with Lyme arthritis. *N Engl J Med.* 1994;330:229-234.

35. Schmidli J, Hunziker T, Moesli P, Schaad UB. Cultivation of *Borrelia burgdorferi* from joint fluid three months after treatment of facial palsy due to Lyme borreliosis. [letter]. *J Infect Dis.* 1988;158:905-906.

36. Preac Murşic V, Marget W, Busch U, Pleterski Rigler D, Hagl S. Kill kinetics of *Borrelia burgdorferi* and bacterial findings in relation to the treatment of Lyme borreliosis. *Infection.* 1996;24:9-16.

37. Liegner KB, Garon G, Dorward D. Lyme borreliosis (LB) studied with the Rocky Mountain Laboratory (RML) antigen capture assay in urine [abstract 18]. Program and abstracts of the Fifth International Conference on Lyme Borreliosis, Arlington, VA, May 30-June 2, 1992. Bethesda, MD: Federation of American Societies for Experimental Biology, 1992.

38. Liegner KB, Agricola MD, Bayer ME, Duray PH. Chronic Lyme disease (CLD): a costly dilemma [abstract P912M]. Program and abstracts of the Sixth International Conference on Lyme Borreliosis. Bologna, Italy, June 19-22, 1994.

39. Liegner KB, Ziska M, Agricola MD, Hubbard JD, Klempner MS, Coyle PK, Bayer ME, Duray PH. Fatal chronic meningoencephalomyelitis (CMEM) with massive hydrocephalus in a New York State patient with evidence of *Borrelia burgdorferi* (*Bb*) exposure [abstract P041T]. Program and abstracts of the Sixth International Conference on Lyme Borreliosis. Bologna, Italy, June 19-22, 1994.

40. Coyle PK, Deng Z, Schutzer SE, Belman AL, Benach J, Krupp LB, Luft B. Detection of *Borrelia burgdorferi* antigens in cerebrospinal fluid. *Neurology.* 1993;43:1093-1097.

41. Coyle PK, Schutzer SE, Deng Z, Krupp LB, Belman AL, Benach JL, Luft BJ. Detection of *Borrelia burgdorferi*-specific antigen in antibody-negative cerebrospinal fluid in neurologic Lyme disease. *Neurology.* 1995;45:2010-2015.

42. Oksi J, Uksila J, Margamaki M, Nikoskelainen J, Viljanen MK. Antibodies against whole sonicated *Borrelia burgdorferi* spirochetes, 41-kilodalton flagellin, and P39 protein in patients with PCR- or culture-proven late Lyme borreliosis. *J Clin Microbiol.* 1995;33:2304-2315.

43. Liegner KB. Lyme disease: the sensible pursuit of answers [guest commentary]. *J Clin Microbiol.* 1993;31:1961-1963.

44. Liegner KB. B. burgdorferi—seek and ye shall find. Expanding the envelope. [guest editorial]. *J Spirochetal Tick-borne Dis.* 1994;1:79-81.

45. Liegner KB. Chronic persistent infection and chronic persistent denial of chronic persistent infection in Lyme disease. Presented at the Lyme Disease Foundation 6th International Conference on Lyme Disease and other Tick-borne Diseases, Atlantic City, NJ, May 5-6, 1993.

46. Lopez-Andreu JA, Ferris J, Canosa CA, Sala-Lizarraga JV. Treatment of late Lyme disease: a challenge to accept [letter]. *J Clin Microbiol.* 1994;32:1415-1416.

47. Liegner KB. Treatment of late Lyme disease, Atlantic City, NJ: a challenge to accept [reply to letter]. *J Clin Microbiol.* 1995;32:1416.

48. Liegner KB, Ganong RB, Heaney D. Rhabdomyolysis occurring during prolonged intravenous antibiotic therapy with ceftriaxone in a patient with Lyme encephalopathy [abstract D624]. VII International Congress on Lyme Borreliosis. San Francisco, CA, June 16-21, 1996.
